# In-Depth Sulfhydryl-Modified Cellulose Fibers for Efficient and Rapid Adsorption of Cr(VI)

**DOI:** 10.3390/polym14071482

**Published:** 2022-04-06

**Authors:** Wenxuan Wang, Feihan Yu, Zhichen Ba, Hongbo Qian, Shuai Zhao, Jie Liu, Wei Jiang, Jian Li, Daxin Liang

**Affiliations:** 1 Key Laboratory of Bio-Based Material Science and Technology (Ministry of Education), Northeast Forestry University, Harbin 150040, China; wxwang@nefu.edu.cn (W.W.); yufeihan@nefu.edu.cn (F.Y.); bazc_@nefu.edu.cn (Z.B.); 1961026882@nefu.edu.cn (H.Q.); zs861944571@163.com (S.Z.); lijiangroup@163.com (J.L.); 2 Department of Military Facilities, Army Logistics Academy, Chongqing 401331, China; liujiely@homail.com; 3 State Key Laboratory of Bio-Fibers and Eco-Textiles, Qingdao University, Qingdao 266071, China; wejiangqd@qdu.edu.cn

**Keywords:** cellulose fibers, adsorption, Cr(VI), sulfhydryl-modified

## Abstract

As one of the hazardous heavy metal ion pollutants, Cr(VI) has attracted much attention in the sewage treatment research field due to its wide distribution range and serious toxicity. In this paper, cellulose fibers were prepared by wet spinning and followed by freeze drying, resulting in large porosity. Subsequently, in-depth sulfhydryl modification was applied with cellulose fibers for efficient and rapid adsorption of Cr(VI). The maximum adsorption capacity of sulfhydryl-modified cellulose fibers to Cr(VI) can reach 120.60 mg g^−1^, the adsorption equilibrium can be achieved within 300 s, and its adsorption rate can reach 0.319 mg g^−1^ s^−1^. The results show that the in-depth sulfhydryl-modified cellulose fibers perform excellent adsorption capacity for chromium, and are also available for other heavy metal ions. At the same time, the low cost and environmentally friendly property of the as-synthesized material also demonstrate its potential for practical usage for the treatment of heavy metal ion pollution in waste water.

## 1. Introduction

Chromium (Cr) is commonly used in tanning, metalworking, mining, and electroplating industries, causing serious pollution of water, soil, and plant resources worldwide [1,2]. Cr in industrial wastewater is mainly hexavalent compounds, such as chromate ions; Cr(VI) toxicity is approximately 100 times higher than Cr(Ⅲ), and it can cause lung cancer and nasopharyngeal cancer [3]. The World Health Organization (WHO) stipulates that the maximum allowable value of Cr(VI) in drinking water is 50 μg/L [4]. Traditional treatment methods, such as membrane separation [5], chemical reduction [6], adsorption [7,8], as well as plasma method [9], have been used to address Cr(VI) in wastewater. Currently, many materials (such as graphene, MXene, microalgal, etc.) are used in the field of adsorption, which is a method with the advantages of reusability, high selectivity, and simple application [10,11,12]. Unfortunately, several problems are associated with these traditional adsorbents, such as low removal efficiency, weak selectivity, and high cost. Therefore, fabricating an excellent adsorbent to remove Cr(VI) in wastewater has become inevitable.

Cellulose is the most widespread biomass material in nature, which is nontoxic, nonpolluting, and easily degraded, making it an environmentally friendly material [13,14]. The dissolution and regeneration of cellulose are necessary for the wide application of cellulose, and different forms of cellulose materials (cellulose microspheres, cellulose fibers, cellulose films, cellulose aerogels, foams, etc.) can be prepared through different solvent systems [15]. The ionic liquid is an excellent cellulose dissolving system, which makes the reshaping of cellulose easy to operate. However, the ionic liquid is expensive and not suitable for large-scale applications. Fortunately, ionic liquids can be recycled and then cellulose dissolved, which greatly reduces the cost [16]. Cellulose fiber (CF) is a material with the advantages of large specific surface area, low density, high mechanical strength, reticular porosity, good hydrophilicity, etc., making it a new type of functional material that is easy to be modified [17]. CFs can be prepared by wet spinning, and pure cellulose fibers also have a certain adsorption effect [18]. Many studies have been carried out on the modification of cellulose fibers to further improve their adsorption efficiency. Song et al. [19] used polyethyleneimine-cellulose fibers for the fast recovery of Au(I) from alkaline e-waste leachate, and Ali et al. [20] used cellulose fiber yarns with high wet strength and their affinity for chitosan combined with the immobilization of tempo-oxidized cyclodextrins to capture 17α-vinyl estradiol from aqueous solutions. These modification methods can improve the adsorption performance of cellulose fibers; unfortunately, the methods are too complex to be implemented and too costly for large-scale applications. Sulfhydryl (–SH) is a common adsorption group in pollutant treatment [21]. Cellulose contains many hydroxyls, and thioglycolic acid is used to esterify it to achieve sulfhydrylation [22]. The adsorption performance of sulfhydryl-modified cellulose is greatly improved, and it can adsorb inorganic/organic pollutants.

Here, we proposed a simple and effective strategy for preparing CFs with a stable 3D network structure using dissolution, regeneration, wet spinning, and freeze drying. Based on the rich pore structure of CFs, thioglycolic acid was used to deeply sulfhydryl-modified them to obtain sulfhydryl-modified cellulose fibers (CFs–SH). The adsorption of Cr(VI) on the CFs–SH has the characteristics of high adsorption capacity and high adsorption efficiency because of being deeply sulfhydryl-modified; simultaneously, the CFs–SH can adsorb more kinds of metal ions, making CFs–SH have a wide application space.

## 2. Materials and Methods

### 2.1. Materials

All chemicals were used without further purification. Potassium dichromate (K_2_Cr_2_O_7_, 99%), sodium hydroxide (NaOH, 99%), cobalt nitrate (Co(NO_3_)_2_•6H_2_O, 99%), cadmium nitrate (Cd(NO_3_)_2_•4H_2_O, 99%), thioglycolic acid (C_2_H_4_O_2_S, 99.5%), diphenylcarbazide (C_13_H_14_N_4_O, 99.7%) and 1-Butyl-3-methyl imidazole chloride ([Bmim]Cl 99%) were obtained from Sigma-Aldrich Co. Ltd. (Shanghai, China). Cupric nitrate (Cu(NO_3_)_2_•3H_2_O, 99%), nickel nitrate (Ni(NO_3_)_2_•6H_2_O, 99%), zinc nitrate (Zn(NO_3_)_2_•6H_2_O, 99%) and lead nitrate (Pb(NO_3_)_2_, 99%) were purchased from Tianjin Kemiou Chemical Reagent Co., Ltd. (Tianjin, China). Hydrochloric acid (HCl, 37%), nitric acid (HNO_3_, 37%), and ethanol (C_2_H_6_O, 99%) were sourced from a local supplier.

### 2.2. Characterization

The morphology and structural characteristics of samples were observed using a scanning electron microscope equipped with an energy-dispersive X-ray spectrometer (SEM-EDS; TM3030, Tokyo Prefecture, Japan). Thermal stability was evaluated using a simultaneous thermal analyzer (Netzsch STA 449F3, Frankfurt, Main, Germany) based on the analysis of thermal gravimetric analysis (TG) and differential scanning calorimetry (DSC), at a temperature range of 50–600 °C with a heating rate of 10 °C min^−1^ under N_2_ atmosphere. The crystal structures of pure CFs and CFs–SH were analyzed using an X-ray diffractometer (XRD; D/max-2200VPC, Tokyo Prefecture, Japan). Fourier transform infrared spectroscopy (FTIR; Frontier, Perkin Elmer, Waltham, MA, USA) was used to characterize the abundant functional group in the pure CFs and CFs–SH. The Cr(VI) concentration was monitored using a UV–vis spectrophotometer (UV-1800, Shimadzu, Kyoto Prefecture, Japan) at 540 nm. The adsorption capacity of CFs-SH toward the adsorption of other heavy metal ions by inductively coupled plasma mass spectrometry (ICP-MS; NEXILN350D, PerkinElmer, Waltham, MA, USA).

### 2.3. Preparation of Pure CFs and CFs–SH

The α-Cellulose (5 wt%) was initially dissolved in [Bmim]Cl at 90 °C for 1 h, the cellulose solution was vacuumed at 80 °C to remove air bubbles, and the solution was injected into ethanol using a 10 mL syringe (needle diameter 800 μm) under the action of a syringe pump. The obtained fibers were washed with water and then prefrozen at −12 °C for 12 h and subsequently freeze-dried for 48 h under high-vacuum conditions (0.010 mbar) at −56 °C to obtain pure CFs. Pure CFs (5 g) were further immersed in thioglycolic acid solution (200 mL, the volume ratio of H_2_SO_4_ and thioglycolic acid was 1:100) for 4 h, and washed 3–5 times in distilled water until the pH of solution was neutral, then finally, dried at 45 °C for 24 h.

### 2.4. Adsorption of Cr(VI)

The dried pure CFs and CFs–SH (50 mg, the optimal adsorbent quality selection was shown in Figure S1) were immersed in 20 mL of K_2_Cr_2_O_7_ aqueous solution (200 mg L^−1^) with stirring at 100 rpm at room temperature (20 ± 1 °C). Pollutants remaining in the filtrate were analyzed in a UV–vis spectrophotometer; the full spectrum before and after adsorption was shown in Figure S2, at a wavelength of 540 nm to test Cr(VI) based on the DPC method [10]. The Cr(VI) adsorption capacity of the adsorbent (*q_e_*) was calculated using Equation (1):

(1) $$q_{e}=\frac{\left(C_{0}-C_{e}\right)V}{m}$$

where *q_e_* is the Cr(VI) equilibrium adsorption capacity, and *C*_0_ (mg L^−1^) and *C_e_* (mg L^−1^) represent the initial and equilibrium concentrations of Cr(VI) in solution, respectively. *V* (L) and *m* (g) refer to the volume of the solution and the mass of samples, respectively.

**Scheme 1:**
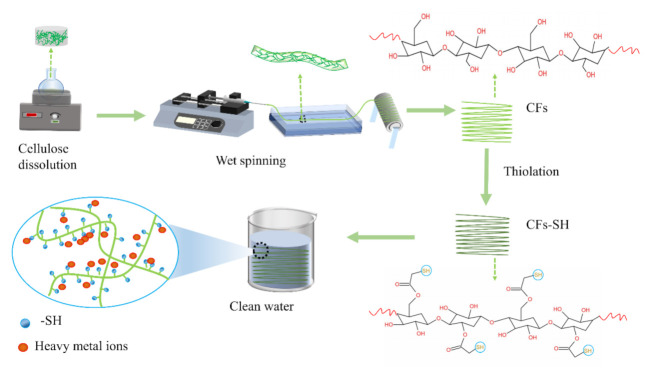
Preparation of in-depth sulfhydryl-modified CFs–SH.

The effects of initial Cr(VI) concentration (5–500 mg L^−1^, the standard curve was shown in Figure S3), pH (1.0, 3.0, 5.0, 7.0, 9.0, 11.0, the optimum pH was shown in Figure S4), and temperature (20–60 °C) on the adsorption process were investigated. All the adsorption experiments were performed in triplicate (n = 3) at least with the mean taken. The flow of the experiment was shown in Scheme 1.

## 3. Test Results and Discussion

### 3.1. Characterization of Pure CFs and CFs–SH

#### 3.1.1. FTIR Analysis

**Figure 1:**
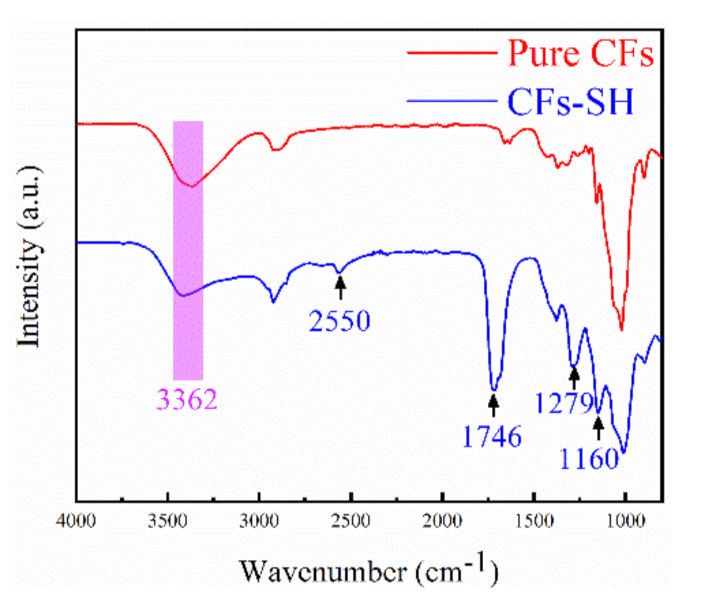
FTIR spectra of pure CFs and CFs−SH.

Figure 1 shows the FTIR spectra of pure CFs and CFs–SH. The FTIR spectra of both aerogels revealed bands at 3362 cm^−1^, which can be attributed to –OH stretching vibration of hydrogen bonds. They play a pivotal role in the process of cellulose dissolution and regeneration, which indicate that the fibers have good hydrophilicity [23,24,25]. The characteristic peak of sulfhydryl (–SH) is 2250 cm^−1^. The C=O stretching vibration peak is 1746 cm^−1^. 1279 cm^−1^ and 1160 cm^−1^ are all the C–O–C stretching vibration peaks. These changes indicate that the esterification reaction between cellulose and thioglycolic acid occurred, resulting in the modification of pure CFs to CFs–SH. 

#### 3.1.2. XRD Analysis

**Figure 2:**
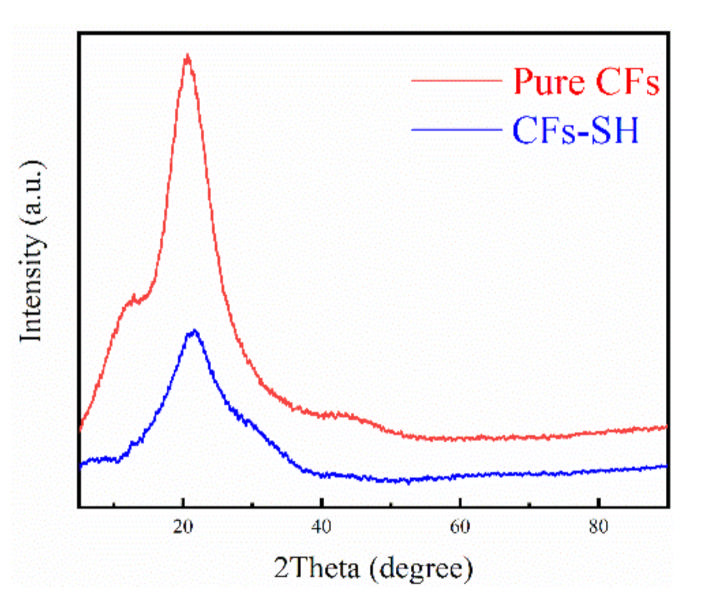
XRD patterns of pure CFs and CFs–SH.

Figure 2 shows the X-ray diffraction patterns of pure CFs and CFs–SH. Note that the crystallinity of CFs–SH decreased compared to pure CFs, this is because the modification of the –SH breaks hydrogen bonds between parts of the cellulose, resulting in a change in crystallinity. At the same time, the mechanical properties of the fibers are reduced (from 155.64 MPa to 69.51 MPa, as shown in Figure S5), which indicates that the modification by –SH was successful.

#### 3.1.3. SEM-EDS Analysis

**Figure 3:**
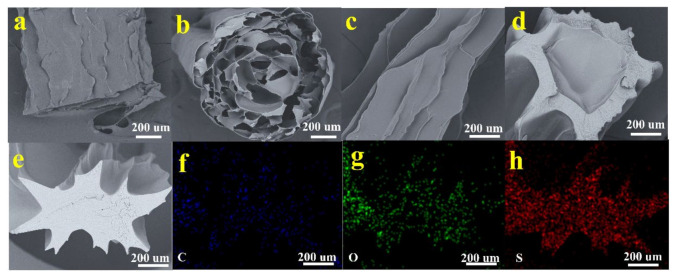
SEM-EDS images of pure CFs and CFs–SH, (**a**) surface of pure CFs, (**b**) cross section of pure CFs, (**c**) surface of CFs–SH, (**d**,**e**) cross section of CFs–SH, (**f**–**h**) EDS mapping of C, O, and S.

The pure CFs obtained by wet spinning and freeze drying have smooth outer walls (Figure 3a) and a rich pore structure inside (Figure 3b), which makes the modification of –SH easier and more thorough. On the contrary, only the surface of the dried CFs can be modified by –SH, and the interior contains almost no sulfur element (Figure S6). The crystallinity of the modified cellulose fibers decreased by –SH, and the porous structure inside the fibers collapsed and stacked (Figure 3c,d). Fortunately, the interior of the CFs has been completely modified, the SEM-EDS cross–section of CFs–SH (Figure 3e–h), so that CFs–SH has a relatively large adsorption capacity.

#### 3.1.4. TG/DTG Analysis

**Figure 4:**
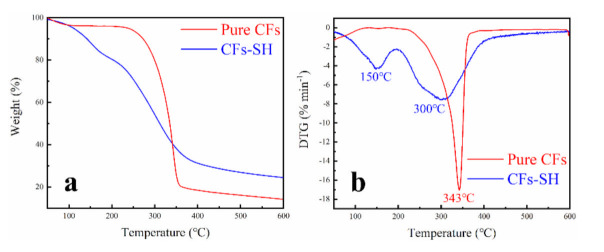
TG (**a**) and DTG (**b**) of pure CFs and CFs–SH.

The thermal stability of pure CFs and CFs–SH were displayed by TG and DTG curves (Figure 4a,b), respectively. Initially, all samples underwent a slight weight loss below 100 °C, belonging to the evaporation of adsorbed or bound water [26,27]. At 343 °C, pure CFs have the maximum decomposition rate, indicating that the decomposition temperature of pure CFs is at 343 °C. At 150 °C, in CFs–SH, the ester group contained in the cellulose successfully modified by thioglycolic acid was thermally decomposed, resulting in a large mass change occurring. The reason why the thermal decomposition temperature of other cellulose (300 °C) was lower than CFs (343 °C) was that the modification reduces the crystallinity of cellulose, which also proved the success of the modification. 

### 3.2. Cr(VI) Adsorption

#### 3.2.1. Effect of Contact Time and Adsorption Kinetics

**Figure 5:**
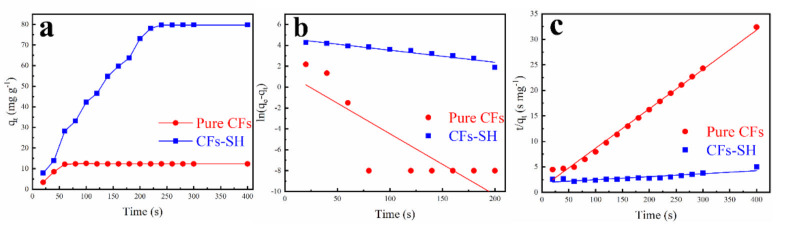
Cr(VI) adsorption behavior in CFs–SH: (**a**) Adsorption kinetics (pH = 3.0; V = 20.0 mL; m ≅ 50 mg; *C*_0_ ≅ 200 mg L^−1^), (**b**) pseudo-first-order kinetics, (**c**) pseudo-second-order kinetics.

Figure 5a shows the adsorption kinetics of the as-prepared pure CFs and CFs–SH. Cr(VI) was rapidly captured by –SH in the CFs–SH within the first two minutes, as shown in the Figure 5a, before achieving the *q_e_* value at 250 s. The adsorption capacity of Cr(VI) is greatly increased by modifying CFs with –SH. Two kinetic models were used to explore the Cr(VI) adsorption behavior in CFs and CFs–SH, in the following equations:

(2) $$\ln\left(q_{e}-q_{t}\right)=\ln q_{e}-k_{1}t$$

(3) $$\frac{t}{q_{t}}=\frac{1}{k_{2}q_{e}^{2}}+\frac{t}{q_{e}}$$

As shown in Figure 5b,c, and Table 1, the fitting results revealed that a pseudo-second-order kinetic model (r^2^ = 0.954) could better describe the adsorption behavior of CFs–SH than a pseudo-first-order one. This indicates that the Cr(VI) adsorption of the adsorbents is mainly dependent on the number of accessible active sites of CFs–SH [28,29,30].

For excellent adsorbents, high adsorption efficiency is critical for future industrial applications [31]. The effect of CFs–SH was confirmed by evaluating the initial Cr(VI) adsorption rate (s) of both adsorbents, following Equation (4).

(4) $$h=k_{2}\times q_{e}^{2}\;t\to 0$$

The adsorption efficiency of CFs–SH is calculated to be 0.319 mg g^−1^ s^−1^, which is due to the large number of adsorption sites brought by the deeply –SH for CFs, which can reach the adsorption equilibrium in a very short time. On the contrary, fibers modified only on the surface layer did not have good adsorption effect (Figure S7). More importantly, the residual Cr(VI) in the treated solution was only 0.03 ± 0.01 mg L^−1^, which met the World Health Organization (WHO) maximum allowable value of Cr(VI) in drinking water [4].

#### 3.2.2. Cr(VI) Adsorption Isotherm on CFs–SH

With Cr(VI) concentrations of 200 mg L^−1^, 0.05 g CFs–SH was added to various 20 mL Cr(VI) solutions. Each sample was left to stand at 20 °C, 30 °C, 40 °C, 50 °C, and 60 °C for 300 s to reach adsorption equilibrium. Subsequently, a preferential analysis was conducted to obtain the final Cr(VI) concentration in the solution. The results were then applied to the Langmuir and Freundlich models to investigate the Cr(VI) adsorption isotherm, using Equations (5) and (6).

(5) $$\frac{C_{e}}{q_{e}}=\frac{C_{e}}{q_{m}}+\frac{1}{q_{m}\times K_{L}}$$

(6) $$\ln q_{e}=\ln K_{F}+\frac{1}{n}\ln C_{e}$$

*q_e_* (mg g^−1^) and *q_m_* (mg g^−1^) are the Cr(VI) adsorption equilibrium capacity and theoretical maximum monolayer Cr(VI) adsorption capacity of CFs–SH, respectively. *K_L_* (L mg^−1^) represents the Langmuir adsorption equilibrium constant. *k_F_* and 1/*n* are the adsorption equilibrium constant and intensity of the concentration effect on adsorption, respectively.

**Figure 6:**
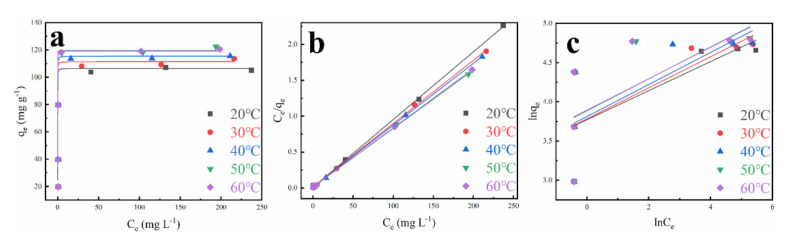
(**a**) Adsorption isotherms, (**b**) Langmuir and (**c**) Freundlich isotherms of Cr(VI) adsorption by CFs–SH at 20 °C, 30 °C, 40 °C, 50 °C and 60 °C (pH = 3.0; V = 20 mL; m ≅ 50 mg; *C*_0_ ≅ 50–500 mg L^−1^).

Figure 6a shows the results of the Cr(VI) adsorption equilibria of CFs–SH. The isotherm parameters were deduced by fitting the data in Figure 6b,c, and these parameters are summarized in Table 2. The adsorption capacity of the adsorbent increases with temperature, which is consistent with the findings reported in the literature. This illustrates that the adsorption process in the adsorbent is an endothermic reaction [32,33].

A comparison of the correlation coefficients (r^2^) indicated that the Langmuir model could better describe the adsorption behavior of the adsorbent, rather than the Freundlich model. This illustrated that similar energy was required during the Cr(VI) adsorption at all sites on CFs–SH, thus confirming the monolayer adsorption of Cr(VI) onto the external and pore surfaces of CFs–SH [34]. The maximum Cr(VI) adsorption capacity of CFs–SH was 120.60 mg g^−1^ (Table 2).

#### 3.2.3. Adsorption Ability of CFs–SH 

Cyclic adsorption/desorption performance was analyzed to investigate the durable performance in terms of performance consistency and the structural stability of CFs-SH and the importance of loading. Unfortunately, after one cycle, the adsorption performance of CFs–SH dropped sharply to 12.49% by the fifth cycle (shown in Figure S8), but it was sufficient as an inexpensive adsorption material.

A comparison of the Cr(VI) adsorption performance by the as-prepared adsorbent relative to that of other composites is summarized in Table 3. CFs–SH exhibited relatively high adsorption capacity; although the adsorption capacity does not have a great advantage, its adsorption rate reaches 0.319 mg g^−1^ s^−1^, which is very important for applications.

### 3.3. Other Heavy Metal Ions Adsorption

**Figure 7:**
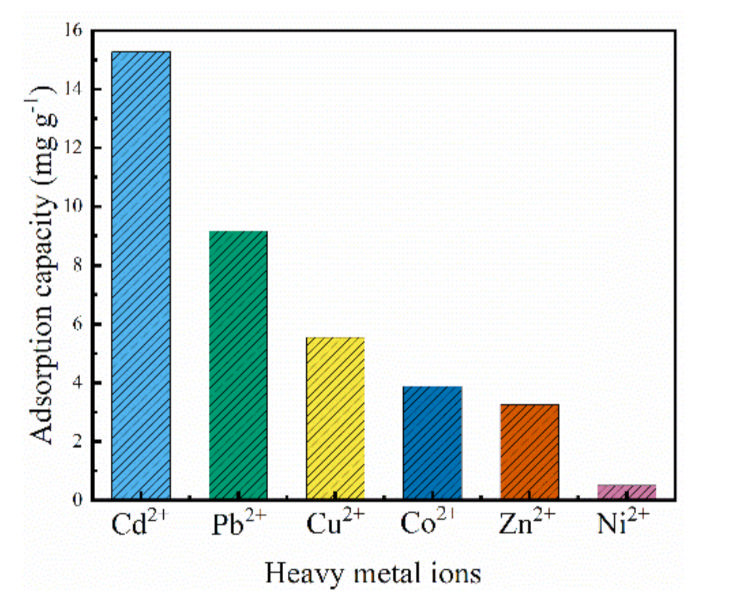
Other heavy metal ions’ adsorption behavior in CFs–SH, including Cd^2+^, Pb^2+^, Cu^2+^, Co^2+^, Zn^2+^, and Ni^2+^ (pH = 3.0; V = 20.0 mL; m ≅ 50 mg; *C*_0_ ≅ 200 mg L^−1^).

In previous studies, –SH also had an adsorption effect on other heavy metal ions [37]. To broaden the application of CFs–SH, six different heavy metal ions (Cd^2+^, Pb^2+^, Cu^2+^, Co^2+^, Zn^2+^, and Ni^2+^) were used to test their adsorption properties. As shown in Figure 7, CFs–SH adsorbs 20 mL, 200 mg L^−1^ different heavy metal ion solutions for 300 s under 20 °C. The adsorption efficiency of CFs–SH was relatively low. The adsorption and adsorption efficiency displayed by CFs–SH is relatively low, but it shows that it has an adsorption effect on various heavy metal ions, which is more promising.

## 4. Conclusions

In this study, CFs–SH with porous structure CFs was deeply modified by sulfhydryl. Therefore, CFs-SH possessed a large number of adsorption sites, which made it become an adsorbent with extremely strong adsorption activity for Cr(VI) and other heavy metal ions. The maximum Cr(VI) equilibrium adsorption capacity of CFs–SH was 120.60 mg g^−1^ at 50 °C, and the Cr(VI) adsorption isotherm agreed well with the Langmuir model. More importantly, CFs–SH could reach the adsorption equilibrium within 300 s, the adsorption rate could reach 0.319 mg g^−1^ s^−1^ at 20 °C, and CFs–SH had certain reusability, which was very useful for practical applications. For future research, improving the reusability of CFs–SH and expanding its adsorption capacity will be the primary task, followed by the improvement of the adsorption efficiency of other heavy metal ions to more effectively remove heavy metal pollution in water.

## Figures and Tables

**Table 1:** Pseudo first order and Pseudo second order.

Material	Pseudo First Order			Pseudo Second Order		
	*q_e_* (mg g^−1^)	*k*_1_ × 10^−3^ (min^−1^)	r^2^	*q_e_* (mg g^−1^)	*k*_1_ × 10^−3^ (min^−1^)	r^2^
Pure CFs	12.34	58.52	0.672	11.49	77.20	0.992
CFs–SH	79.73	11.55	0.906	78.32	5.86	0.954

**Table 2:** Langmuir model and Freundlich model.

Temperature	Langmuir Isotherm			Freundlich Isotherm		
	*q_m_* (mg g^−1^)	*k*_1_ × 10^−3^	r^2^	*k*_2_	*n*	r^2^
20 °C	105.08	9.42	0.999	0.185	3.77	0.471
30 °C	113.57	8.80	0.999	0.198	3.78	0.491
40 °C	115.58	8.61	0.999	0.205	3.81	0.470
50 °C	122.51	8.15	0.999	0.203	3.88	0.393
60 °C	120.60	8.24	0.999	0.200	3.89	0.376

**Table 3:** Comparison of the Cr(VI) adsorption performance of CFs–SH with that of other composites.

Composites	Adsorption Capacity (mg g^−1^)	Adsorption Rate (mg g^−1^ s^−^^1^)	Ref.
Peanut shell biochar	22.93	0.013	[4]
HI	353.87	0.098	[11]
SBA-15	66.50	0.004	[21]
PAIN/TiO_2_	394.43	0.219	[35]
Brazilian-pine fruit coat	240.00	0.008	[36]
CFs–SH	120.60	0.319	This study

## Data Availability

The data presented in this study are available in the manuscript and Supplementary Material.

## Supplementary Materials

The following supporting information can be downloaded at: https://www.mdpi.com/article/10.3390/polym14071482/s1, Figure S1: Standard curve of Cr(VI), Figure S2: The full UV-Vis spectrum of different solution. Figure S3. Standard curve of Cr(VI). Figure S4. Adsorption rate under different pH conditions. Figure S5. The stress-strain curve of pure CFs and CFs–SH. Figure S6. SEM–EDS images of cellulose fibers after drying at 50 °C. Figure S7. The adsorption performance of cellulose fibers after drying at 50 °C. Figure S8. Cyclic adsorption/desorption performance of CFs–SH.
