# Supplementary material for: In-Depth Sulfhydryl-Modified Cellulose Fibers for Efficient and Rapid Adsorption of Cr(VI)
Source: Polymers (Basel). 2022 Apr 6;14(7):1482. doi: 10.3390/polym14071482 (PMC9002529; doi:10.3390/polym14071482)
Supplement: Supplementary file 1 [file polymers-14-01482-s001.zip › polymers-1637853-supplementary.pdf]

Supplementary Information

In-Depth Sulfhydryl-Modified Cellulose Fibers for Efficient and Rapid Adsorption of Cr(VI)

Wenxuan Wang [1], Feihan Yu [1], Zhichen Ba [1], Hongbo Qian [1], Shuai Zhao [1], Jie Liu [2], Wei Jiang [3], Jian Li [1] and Daxin Liang [1,*]

[1] Key Laboratory of Bio-based Material Science and Technology (Ministry of Education), Northeast Forestry University, Harbin 150040, China; wxwang@nefu.edu.cn (W.W.); yufeihan@nefu.edu.cn (F.Y.); bazc_@nefu.edu.cn (Z.B.); 1961026882@nefu.edu.cn (H.Q.); zs861944571@163.com (S.Z.); lijiangroup@163.com (J.L.)

[2] Department of Military Facilities, Army Logistics Academy, Chongqing 401331, China; liujiely@homail.com (J.L.)

[3] State Key Laboratory of Bio-Fibers and Eco-Textiles, Qingdao University, Qingdao 266071, China; wejiangqd@qdu.edu.cn (W.J.)

* Correspondence: daxin.liang@nefu.edu.cn; Tel.: +86-18182806182

The optimum adsorbent quality was optimized by adding 0.01 g, 0.02 g, 0.03 g, 0.04 g, 0.05 g, 0.06 g and 0.07 g CFs–SH to 20 mL, 200 mg L^{-1} Cr(VI) solution respectively, testing after 200 s, 50 mg CFs–SH was the best quality as shown in Figure S1.

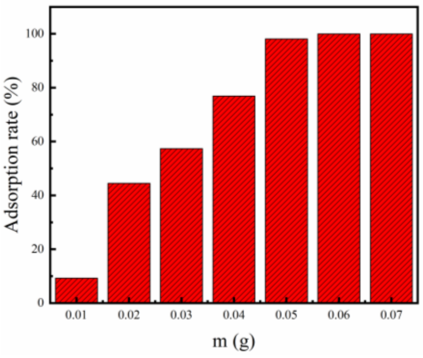

Figure S1. Adsorption rate under different quality of CFs–SH.

The full UV-Vis spectrum of 200 mg L^{-1} Cr(VI), 0 mg L^{-1} and 200mg L^{-1} Cr(VI) after adsorption are shown in Figure S2.

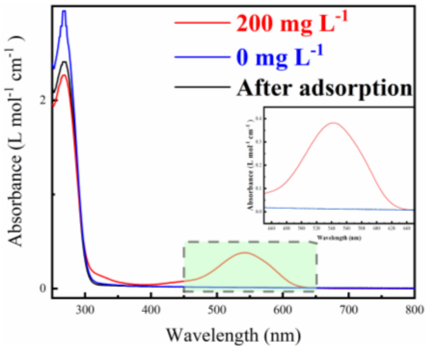

Figure S2. The full UV-Vis spectrum of different solution.

A standard curve was established by the relationship between different concentrations of chromium solution and absorbance, Standard formula Equation (S1):

Equation (S1)

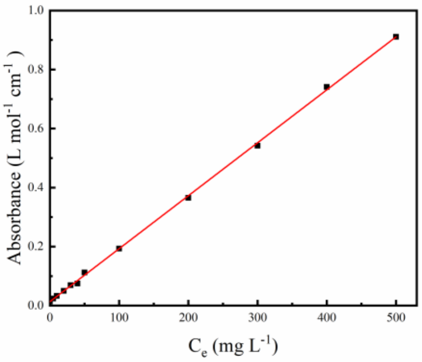

Figure S3. Standard curve of Cr(VI).

The adsorption efficiency of Cr(VI) (20 mL, 200mg L^{-1}) was explored using 0.1 M NaOH and HCl to adjust different pH. The degradation efficiencies at pH = 1, 3, 5, 7, 9, 11, 13 were shown in Figure S2. The optimum pH is 3.0, and the following experiments use this condition.

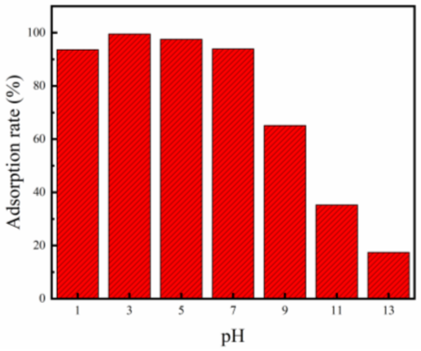

Figure S4. Adsorption rate under different pH conditions.

The mechanical properties of CFs decreased from 155.64 MPa to 69.51 MPa due to the reduction of the crystallinity of cellulose by the modification of sulfhydryl groups

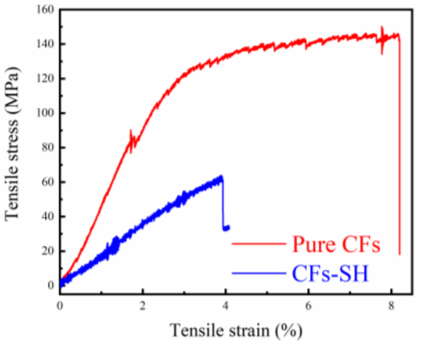

Figure S5. The stress-strain curve of pure CFs and CFs-SH.

SEM-EDS image of cellulose fibers after drying at 50°C and modified with thioglycolic acid.

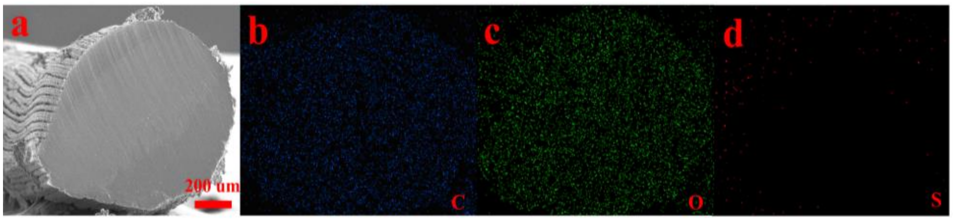

Figure S6. SEM-EDS images of cellulose fibers after drying at 50°C.

The adsorption efficiency of cellulose fibers after drying at 50°C was explored (pH = 3.0; V = 20.0 mL; m ≅ 50 mg; C_0 ≅ 200 mg L^{-1}).

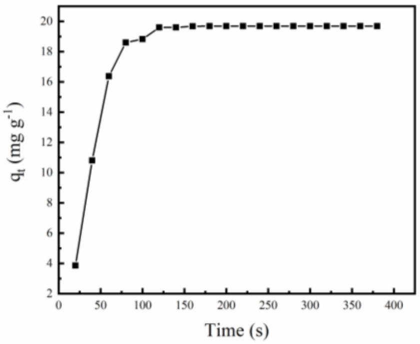

Figure S7. The adsorption performance of cellulose fibers after drying at 50°C.

The cyclic adsorption performance of CFs-SH was explored. The elution of Cr(VI) was carried out by HCl (10%) and EDTA (1 mol L^{-1}), and the cyclic experiment was carried out after washing and drying.

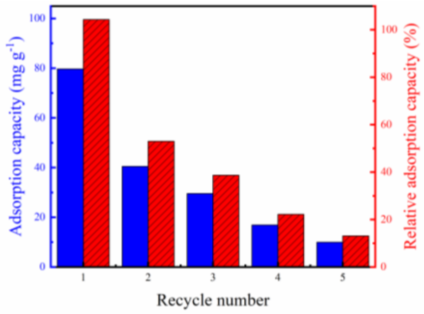

Figure S8. Cyclic adsorption/desorption performance of CFs-SH (pH = 3.0; V = 20.0 mL; m ≅ 50 mg; C_0 ≅ 200 mg L^{-1}).
